# LncRNA VPS9D1-AS1 promotes cell proliferation in acute lymphoblastic leukemia through modulating GPX1 expression by miR-491-5p and miR-214-3p evasion

**DOI:** 10.1042/BSR20193461

**Published:** 2020-10-05

**Authors:** Shishan Xiao, Na Xu, Qian Ding, Shengwen Huang, Yan Zha, Hongqian Zhu

**Affiliations:** 1 Department of Hematology, Guizhou Provincial People’s Hospital, Guiyang 550002, Guizhou, China; 2 Department of Hematology, Nanfang Hospital, Southern Medical University, Guangzhou 510515, Guangdong, China

**Keywords:** acute lymphoblastic leukemia (ALL), glutathione peroxidase 1 (GPX1), miR-214-3p, miR-491-5p, VPS9D1 antisense RNA 1 (VPS9D1-AS1)

## Abstract

Alterations in messenger RNAs (mRNAs) of protein-coding genes can influence the malignant behaviors of acute lymphoblastic leukemia (ALL) cells. According to the prediction from The Cancer Genome Atlas (TCGA) database, we discovered that glutathione peroxidase 1 (GPX1) was up-regulated in acute myeloid leukemia (LAML) tissues, which pushed us to explore the feasible role and its related modulatory mechanism of GPX1 in ALL. In this research, we first proved the high expression of GPX1 in ALL cells compared with normal cells. Functional assays further revealed that the proliferation was obstructed and the apoptosis was facilitated in ALL cells with silenced GPX1. Then, both miR-491-5p and miR-214-3p that were down-regulated in ALL cells were affirmed to target GPX1. Subsequently, VPS9D1 antisense RNA 1 (VPS9D1-AS1) was recognized as the upstream regulator of miR-491-5p-miR-214-3p/GPX1 axis in a competing endogenous RNA (ceRNA) model. Importantly, we proved that VPS9D1-AS1 served as a tumor promoter in ALL through elevating GPX1. In conclusion, VPS9D1-AS1 contributed to ALL cell proliferation through miR-491-5p-miR-214-3p/GPX1 axis, hinting an optional choice for the treatment of ALL.

## Introduction

Acute lymphoblastic leukemia (ALL) is defined as a hematologic malignancy that originated from the precursors of the lymphoid lineage, and it is one of the most frequent cancers happening in the youth, especially children [1–3]. To better understand the pathology and therapy of ALL, we are committed to exploring novel biomarkers for ALL patients.

MicroRNAs (MiRs) are short single-stranded RNAs with approximately 18–25 nucleotides (nt). Recently, miRs are reported to exert diverse functions in cancer by affecting gene expression at post-transcriptional level [4,5]. For instances, up-regulated miR-186-5p inhibits cell proliferation, metastasis and epithelial-to-mesenchymal transition (EMT) in colorectal cancer via targeting ZEB1 [6]; miR-612 represses stem cell-like property in hepatocellular carcinoma by controlling Sp1/Nanog pathway [7]; miR-378 enhances cell survival, tumor growth and angiogenesis through targeting SuFu and Fus-1 [8]; miR-375-HOXB3-CDCA3/DNMT3B regulatory loop makes a contribution to leukemogenesis in acute myeloid leukemia (LAML) [9].

As past researches mentioned, miR-491-5p and miR-214-3p have inhibitory effects on cancer progression [10–12]. MiR-491-5p suppresses cell proliferation and invasion via inhibition of IGF2BP1 in non-small cell lung cancer [13]. MiR-491-5p restrains cell proliferative, invasive and migratory abilities via targeting JMJD2B in gastric cancer [14]. Increased miR-214-3p enhances the cisplatin-sensitivity of esophageal squamous cancer cells by targeting survivin and CUG-BP1 [15]. However, the participation of these two miRs in ALL has never been explored.

In competing endogenous RNA (ceRNA) network, long noncoding RNAs (lncRNAs) sponge miRs to release messenger RNAs (mRNAs) from RNA-induced silencing complexes (RISCs) [16,17]. For example, FER1L4/miR-372/E2F1 pathway regulates cell proliferation and cell cycle progression in glioma [18]; LINC00665 promotes lung adenocarcinoma development by serving as an miR-98 sponge to regulate AKR1B10-ERK pathway [19]; lncRNA GAS5-miR-23a-ATG3 axis regulates autophagy in breast cancer [20]. LncRNA VPS9D1 antisense RNA 1 (VPS9D1-AS1) is indicated to promote prostate cancer cell proliferation by targeting miR-184/c-Myc pathway and to serve as a prognosis predictor in non-small cell lung cancer [21,22]. Nonetheless, whether VPS9D1-AS1 is implicated in the initiation and progression of ALL is an obscure question.

Our study purposed to probe into the detailed molecular mechanism in ALL, so as to found novel biomarkers for ALL treatment. In the present paper, glutathione peroxidase 1 (GPX1) was proved to be overexpressed in ALL and be rescued by VPS9D1-AS1 from miR-491-5p and miR-214-3p induced silencing, resulting in aggravated cell proliferation in ALL.

## Materials and methods

### Cell culture

Human acute lymphoblastic leukemia cell lines (Molt-4 and Molt-3) and human peripheral blood mononuclear cells (PBMCs) were purchased from the American Type Culture Collection (ATCC, Manassas, VA, U.S.A.). All cells were cultured in RPMI-1640 medium (Invitrogen, Carlsbad, CA, U.S.A.) containing 10% FBS (Invitrogen) and 1% penicillin/streptomycin (Sigma–Aldrich, Milan, Italy) in a humidified incubator which was filled with 5% CO_2_ at 37°C.

### Cell transfection

For the knockdown of GPX1 and VPS9D1-AS1, shRNAs (sh-GPX1#1/2, sh-VPS9D1-AS1 and sh-NC) were constructed by Gene Pharma (Shanghai, China). Meanwhile, the miR-491-5p mimics and miR-214-3p mimics that were applied to overexpress miR-491-5p and miR-214-3p were also synthesized by Gene Pharma, with miR-NC as the negative controls. For cell transfection, cells were put into a fresh six-well plate and grown to 80% confluence, and the transfection was conducted using Lipofectamine 2000 (Invitrogen) based on supplier’s guidance. Transfected cells were reaped 48 h later for following assays.

### Quantitative real-time PCR

Quantitative real-time PCR (qRT-PCR) was performed as previously described [23]. In detail, total RNA was extracted from cells via use of TRIzol reagent (Thermo Fisher Scientific, Waltham, MA), followed by reverse transcription into cDNA using a Reverse Transcription Kit (Invitrogen). qRT-PCR analysis was performed by SYBR Green Premix PCR Master Mix (Roche, Mannheim, Germany) with an ABI HT9600 (Applied Biosystems, Foster City, CA, U.S.A.). Relative expression of genes was calculated by 2^−∆∆*C*_t_^ method. GAPDH (for VPS9D1-AS1 and GPX1) or U6 (for miR-491-5p and miR-214-3p) was the control for expression normalization.

### Western blot

Cells were lysed by using RIPA lysis buffer (Beyotime, Shanghai, China) containing protease inhibitor to obtain total proteins. Protein concentrations were assessed under a Bio-Rad DC Protein Assay Kit (Yuwei Biotechnology, Guangzhou, China). After that, proteins were separated by SDS/PAGE and then moved on to the PVDF membrane. The membrane was sealed tightly with fat-free milk, followed by co-cultured with specific primary antibodies: anti-GPX1 (ab22604, Abcam, Cambridge, U.S.A.), anti-total caspase-3 (ab13847, Abcam), anti-cleaved caspase-3 (ab2302, Abcam), anti-total caspase-9 (ab32539, Abcam), anti-cleaved caspase-9 (ab2324, Abcam) and anti-GAPDH (ab8245, Abcam). After being incubated with secondary antibodies, the membrane was washed, and the protein bands were observed via chemiluminescence detection system and quantified via ImageJ software.

### Cell viability assay

The viability of Molt-3 and Molt-4 cells was assessed by the use of a CCK-8 kit (Dojindo Laboratories, Kumamoto, Japan). The cells were first plated into fresh 96-well plates, followed by cultivation over specific time points. A total of 10 µl of CCK-8 reagent was added to incubate for additional 4 h. Finally, the determination of absorbance at 450 nm was performed with a microplate reader (Bio-Tek Instruments, Hopkinton, MA, U.S.A.).

### EdU incorporation assay

This experiment was performed by EdU incorporation assay kit which was bought from Ribobio (Invitrogen). Transfected cells were placed into 96-well plates and cultivated with 100 μl EdU medium diluent for 3 h. Cells were incubated with 0.5% Troxin X-100 before cells were fixed in 4% paraformaldehyde (Solarbio, Beijing, China) and treated with 1× Apollo® 488 fluorescent staining solution. DAPI was employed to dye the nuclei and the images of cells were captured via a fluorescent microscope (Olympus, Tokyo, Japan).

### Colony formation assay

After transfection, cells were inoculated into six-well plates with 800 cells per well. Two weeks later, colonies were cleaned in PBS (Solarbio), fixed with the utilization of methanol (Solarbio) and dyed in Crystal Violet (Sigma–Aldrich). The visible colonies were then counted manually.

### Apoptosis assay

Molt-3 or Molt-4 cells were cultured in six-well plates for 48 h and cleaned with PBS (Solarbio). Cells were then resuspended in PBS (Solarbio) and processed with double staining by Annexin V-fluorescein isothiocyanate (FITC) and Propidium Iodide (PI). Afterward, the rate of apoptosis was examined by flow cytometry (Becton, Dickinson and Company).

### Beyotime C115 caspase activity assay

The activities of caspase-3 and caspase-9 in indicated cells were examined using caspase activity kits (Beyotime) strictly in -line with the instructions. In a nutshell, cells were lysed and then cell lysates were gathered in low speed centrifuge. Equal amount of 10-µl proteins from every sample was added into 96-well plates and combined with 80-µl reaction buffer supplemented with caspase substrates. Finally, caspase activities at 405 nm were determined by using TECAN reader (TECAN Commercial, Shanghai, China).

### RNA pull-down assay

The miR-491-5p sense/antisense, miR-214-3p sense/antisense and nonsense control (NC) sequences were biotin-labeled into Bio-miR-491-5p sense/antisense, Bio-miR-214-3p sense/antisense and Bio-NC. The biotinylated RNAs were co-incubated with cell lysates overnight, and then co-cultured with the magnetic beads which were adhered with streptavidin for 4 h. At last, the purified RNAs were detected by qRT-PCR.

### Luciferase reporter assay

The full-length VPS9D1-AS1 or GPX1 3′UTR sequence with wildtype or mutant binding sites for miR-214-3p or miR-491-5p were subcloned into pmirGLO dual-luciferase vector to construct VPS9D1-AS1-WT/Mut and GPX1-WT/Mut vectors, separately. The luciferase activities were determined by Dual-luciferase reporter system.

### RNA immunoprecipitation assay

EZMagna RNA immunoprecipitation (RIP) kit (Millipore, Billerica, MA, U.S.A.) was applied for conducting RIP experiments. Molt-3 or Molt-4 cells were cultured in RIP buffer containing magnetic beads conjugated with human anti-Ago2 antibody (Millipore) or anti-IgG (Millipore). Besides, normal IgG and input were seen as the negative and positive controls, respectively. Moreover, the purified RNAs were subjected to qRT-PCR analysis.

### Subcellular fractionation

Cytoplasmic and nuclear RNAs were separated by using PARIS™ Kit (Ambion, Austin, TX, U.S.A.). A total of 1 × 10^7^ Molt-3 and Molt-4 cells were harvested and re-suspended, followed by lysing via cell fractionation buffer. After centrifugation, the upper solution was gently removed to another tubes to serve as the cytoplasmic fraction. The nuclear pellet was gained and kept to separate RNA by cell disruption buffer. In the end, the expression levels of VPS9D1-AS1, U6 (the nuclear control) and GAPDH (the cytoplasmic control) were analyzed by qRT-PCR.

### FISH assay

Subcellular localization of VPS9D1-AS1 was determined via a FISH Kit (Roche, Basel, Switzerland). Cells were processed with 4% paraformaldehyde (Solarbio) for fixation. Then, a VPS9D1-AS1 probe (Sigma–Aldrich) and digoxigenin included hybridization solution was added into the plate. DAPI (Sigma–Aldrich) was applied for nucleus staining for 10 min. Finally, the fluorescence images were gained by a laser confocal scanning microscopy (Olympus).

### Statistical analysis

Data were shown as mean ± SD. GraphPad Prism 7 software package (La Jolla, CA, U.S.A.) was used for the analysis of all the experimental data. Student’s *t* test and ANOVA were employed for confirming the difference between two or more groups. *P*<0.05 was regarded to have statistical significance. Each experiment was done thrice independently.

## Results

### GPX1 inhibition weakened the proliferation of ALL cells

**Figure 1:**
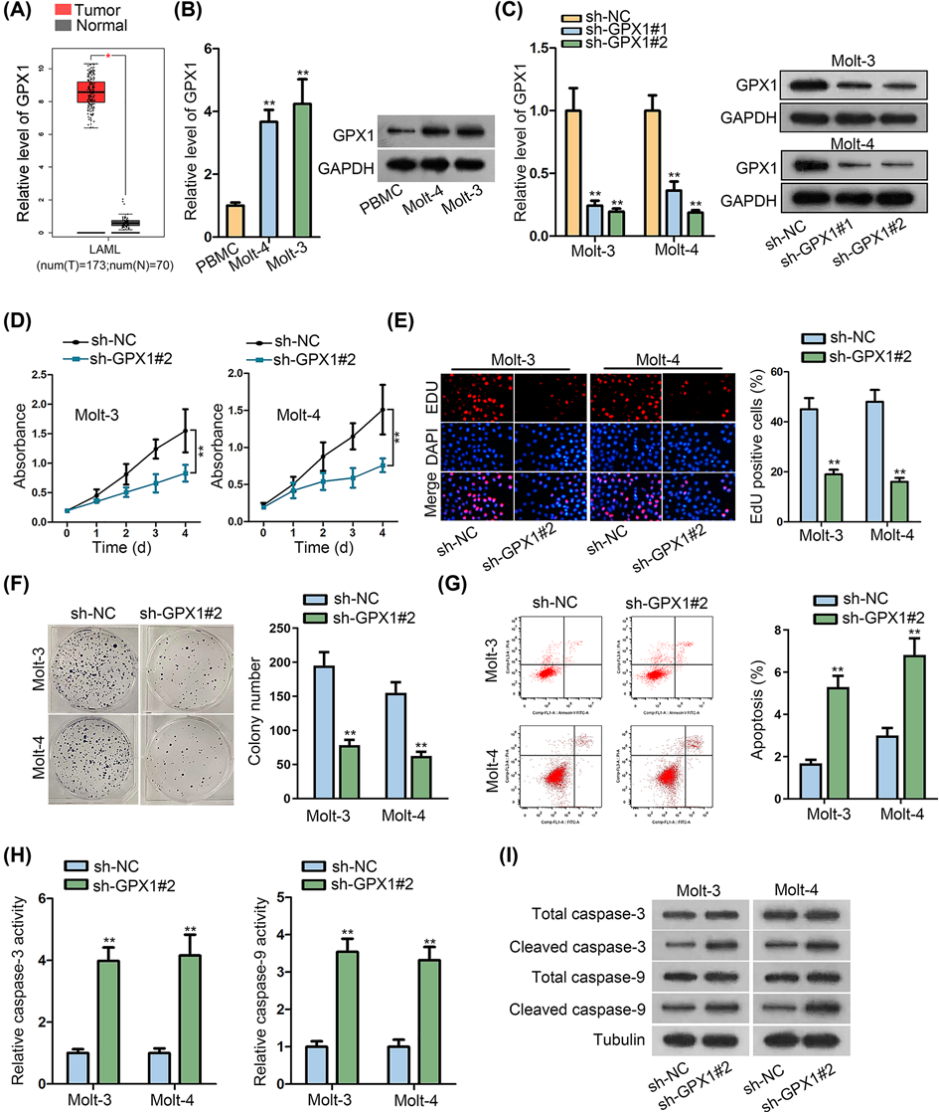
The inhibition of GPX1 depletion on ALL cell proliferation (**A**) The significant up-regulation of GPX1 in LAML tissues from TCGA dataset. (**B**) Both mRNA and protein levels of GPX1 were elevated in ALL cells compared with the normal PBMCs. (**C**) qRT-PCR proved that GPX1 was silenced at mRNA and protein levels in Molt-3 and Molt-4 cells. (**D**) CCK-8 assay indicated that ALL cell viability was weakened by silencing GPX1. (**E**) EdU assay results indicated that EdU-positive cell percent was lowered after transfecting cells with sh-GPX1#2. (**F**) GPX1 depletion led to reduced colonies as assessed by colony formation assay. (**G**) Flow cytometry analysis showed that the apoptotic rate of ALL cells was promoted by inhibiting GPX1. (**H**) Caspase-3/9 activity was enhanced in ALL cells responding to GPX1 knockdown. (**I**) Western blotting revealed that depleted GPX1 caused the elevated protein levels of cleaved caspase-3/9. All assays were run in triplicate. **P<0.01.

GPX1 was predicted to be highly expressed in LAML tissues by The Cancer Genome Atlas (TCGA) website (Figure 1A), hence we wondered whether GPX1 played a role in ALL. qRT-PCR and Western blot analyses demonstrated that GPX1 mRNA and protein expression was distinctly up-regulated in ALL cells compared with normal PBMCs (Figure 1B and Supplementary Figure S1A). Subsequently, GPX1 was silenced in Molt-3 and Molt-4 cells via transfecting with sh-GPX1#1 or sh-GPX1#2, as determined by qRT-PCR and Western blot experiments (Figure 1C and Supplementary Figure S1B). The results of CCK-8 demonstrated that the viability of both Molt-3 and Molt-4 cells was repressed with silence of GPX1 (Figure 1D). EdU and colony formation assays indicated that cell proliferation was hindered when GPX1 was down-regulated in these two ALL cells (Figure 1E,F). By contrast, flow cytometry analysis exhibited that cell apoptosis was apparently induced by inhibited GPX1, as more apoptotic cells were observed in Molt-3 and Molt-4 cell populations when GPX1 was decreased (Figure 1G). Meanwhile, we detected the elevated caspase-3/9 activity and the strengthened levels of active caspase-3/9 in two ALL cells under GPX1 inhibition (Figure 1H,I and Supplementary Figure S1C). In other words, suppressing GPX1 restrained cell proliferation but activated cell apoptosis in ALL.

### GPX1 was targeted by miR-491-5p and miR-214-3p in ALL

**Figure 2:**
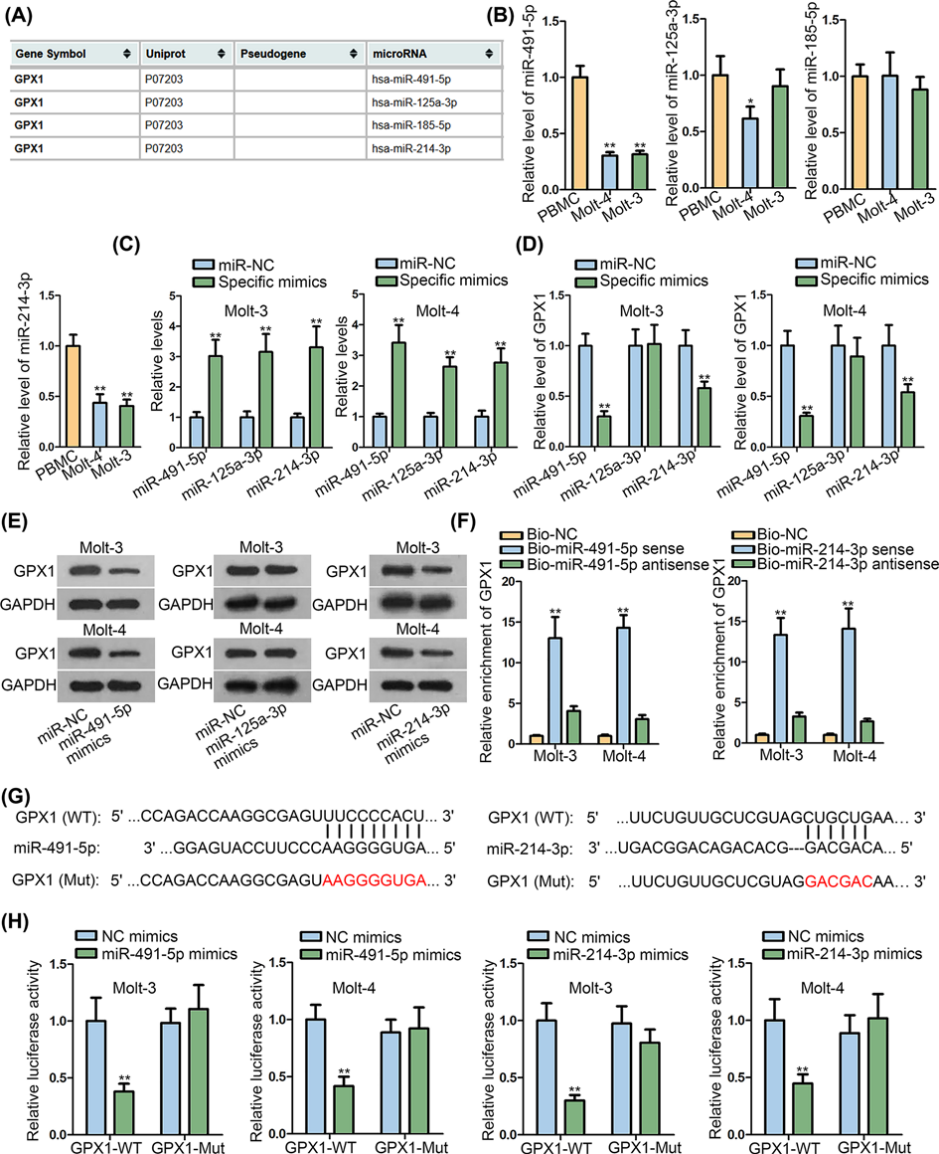
Both miR-491-5p and miR-214-3p targeted GPX1 in ALL cells (**A**) Four miRNAs interacting with GPX1 were predicted by mirDIP. (**B**) The expression of above four miRNAs were detected in ALL cells using qRT-PCR. (**C**) qRT-PCR determined that miR-491-5p, miR-125a-3p and miR-214-3p were overexpressed by specific mimics in ALL cells. (**D,E**) qRT-PCR and Western blot proved that the mRNA and protein levels of GPX1 were decreased by miR-491-5p or miR-214-3p overexpression, but not changed by miR-125a-3p overexpression. (**F**) RNA pull-down assay unveiled that GPX1 was enriched in the RNA complexes pulled-down by Bio-miR-491-5p sense or Bio-miR-214-3p sense. (**G**) The wildtype or mutant binding sites of miR-491-5p or miR-214-3p in GPX1 3′-UTR. (**H**) Luciferase reporter assay detected the interaction between GPX1 and miR-491-5p (or miR-214-3p) in ALL cells. All assays were run in triplicate. *P<0.05 and **P<0.01.

MiRs are reported to exert their functions via binding to the 3′UTR of specific mRNAs to silence the level of these mRNAs. By using mirDIP, we found four miRs possessing very high binding affinities with GPX1 (Figure 2A). Subsequently, we discovered that the expression of miR-491-5p, miR-125a-3p and miR-214-3p presented to be reduced to different degrees in tumor cells, whereas that of miR-185-5p was not changed (Figure 2B). Further, it manifested that GPX1 expression was declined under overexpression of miR-491-5p and miR-214-3p, whereas nearly unaffected by up-regulated miR-125a-3p (Figure 2C–E and Supplementary Figure S1D). Then, RNA pull-down assay unveiled that GPX1 mRNA was specially pulled down by biotinylated miR-491-5p and miR-214-3p (Figure 2F). Thereafter, we acquired the binding sequences between above molecules (Figure 2G). Interestingly, we discovered that the concentration of miR-214-3p which possessed two sites in GPX1 3′UTR was almost two-folds to that of GPX1 in both Molt-3 and Molt-4 cells, while the concentration of miR-491-5p which had only one site in GPX1 3′UTR was slightly higher than that of GPX1 in these two cells (Supplementary File S1A). Importantly, luciferase reporter assay revealed that the luciferase activity of GPX1-WT, but not GPX1-Mut, was evidently weakened in response to improved expression of miR-491-5p or miR-214-3p (Figure 2H). Thereafter, we explored the cellular function of miR-491-5p and miR-214-3p in ALL. Interestingly, in two ALL cells, the ectopic expression of either above two miRs led to declined viability and proliferation (Supplementary Figure S2A–C) and accelerated apoptosis (Supplementary Figure S2D–F). These findings told us that GPX1 was targeted by both miR-491-5p and miR-214-3p, two tumor inhibitors in ALL.

### VPS9D1-AS1 was the upstream regulator of miR-419-5p and miR-214-3p

**Figure 3:**
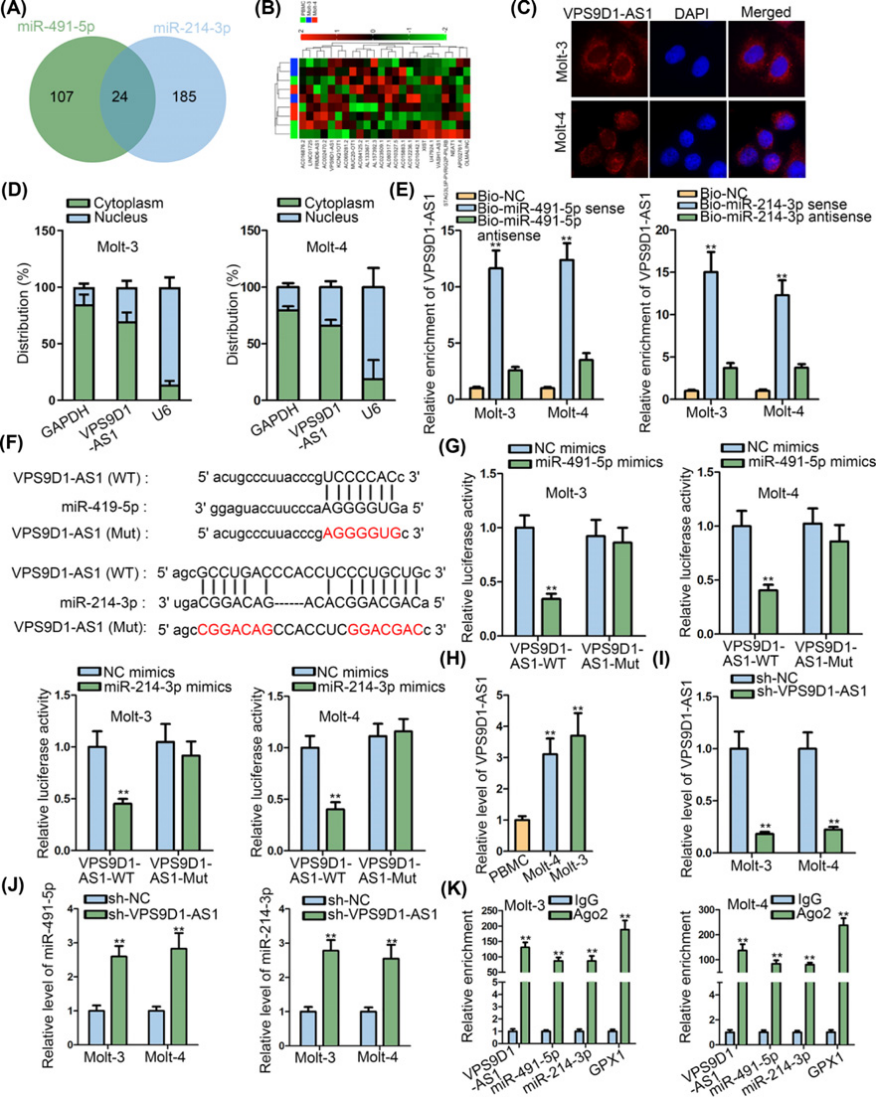
VPS9D1-AS1 sponged miR-491-5p and miR-214-3p in ALL cells (**A**) The 24 lncRNAs shared between miR-491-5p and miR-214-3p were presented in Venn diagram. (**B**) The fold changes of above 24 lncRNAs in ALL cells relative to normal PBMCs were assessed by qRT-PCR. (**C,D**) FISH and subcellular fractionation assays suggested that VPS9D1-AS1 was mainly located in the cytoplasm of ALL cells. (**E**) RNA pull-down assay proved that VPS9D1-AS1 was pulled-down by Bio-miR-491-5p sense or Bio-miR-214-3p sense. (**F**) The interacting sequences between VPS9D1-AS1 and miR-491-5p (or miR-214-3p). (**G**) Luciferase reporter assay unveiled that overexpression of miR-491-5p or miR-214-3p diminished the luciferase activity of VPS9D1-AS1-WT. (**H**) qRT-PCR disclosed that VPS9D1-AS1 was highly expressed in ALL cells. (**I,J**) qRT-PCR validated that the sh-VPS9D1-AS1 transfection repressed VPS9D1-AS1, but strengthened miR-491-5p and miR-214-3p levels in ALL cells. (**K**) RIP assay confirmed that VPS9D1-AS1, miR-491-5p, miR-214-3p and GPX1 were all precipitated by anti-Ago2 antibody. All assays were run in triplicate. **P<0.01.

Considering that lncRNAs can participate in the etiology and progression of cancers via ceRNA networks, we assumed that there might be some lncRNA upstream of miR-419-5p-miR-214-3p/GPX1 axis. Through browsing starBase v3.0, we intersected the potential lncRNAs interacting with miR-491-5p and miR-214-3p, and eventually obtained 24 shared lncRNAs (Figure 3A). Intriguingly, among these 24 lncRNAs, VPS9D1-AS1 was verified as the one with most obvious up-regulation in tumor cells in comparison with normal controls (Figure 3B). Moreover, we revealed that VPS9D1-AS1 was mainly located in the cytoplasm of two ALL cells (Figure 3C,D). Besides, RNA pull-down experiments validated the interaction of both miR-491-5p and miR-214-3p with VPS9D1-AS1 in Molt-3 and Molt-4 cells (Figure 3E). Then, corresponding binding sequences between miR-491-5p (or miR-214-3p) and VPS9D1-AS1 were exhibited in Figure 3F. As expected, the luciferase activity of VPS9D1-AS1-WT, rather than that of VPS9D1-AS1-Mut, was impaired when miR-491-5p or miR-214-3p was overexpressed (Figure 3G). Next, we discovered the overexpression of VPS9D1-AS1 in ALL cells (Figure 3H). Additionally, both the levels of miR-491-5p and miR-214-3p were augmented under VPS9D1-AS1 depletion (Figure 3I,J). Moreover, the high enrichment of VPS9D1-AS1, miR-491-5p, miR-214-3p and GPX1 in Ago2 group was viewed in both Molt-3 and Molt-4 cells (Figure 3K). These data exposed that VPS9D1-AS1 served as a ceRNA against GPX1 through absorbing miR-491-5p and miR-214-3p.

### VPS9D1-AS1 influenced cell proliferation and apoptosis via GPX1 in ALL

**Figure 4:**
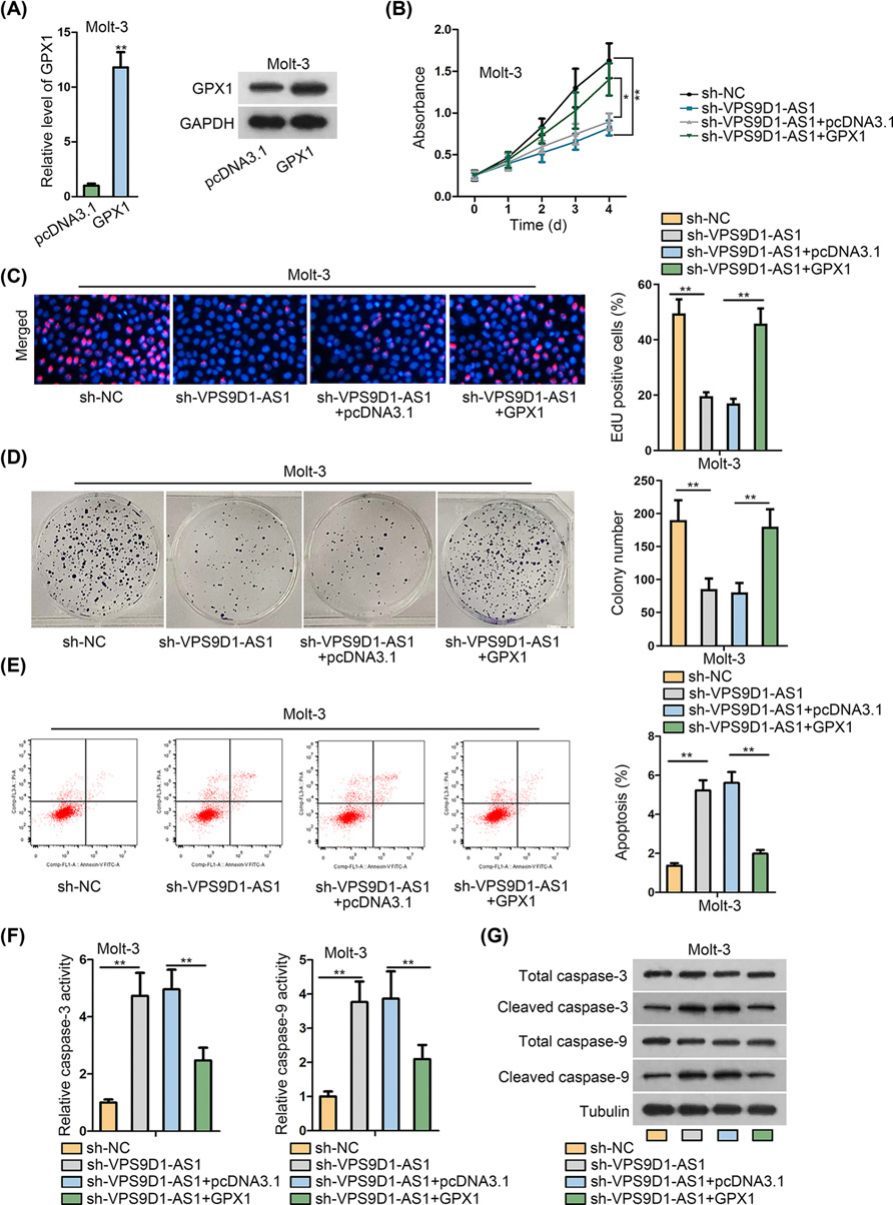
VPS9D1-AS1 promoted ALL cell proliferation through GPX1 (**A**) Transfection of the specific pcDNA3.1 vector containing GPX1 cDNA sequence increased the mRNA and protein levels of GPX1 in Molt-3 cells, as verified by qRT-PCR. (**B**–**D**) The proliferation ability of Molt-3 cells was inhibited by VPS9D1-AS1 depletion, but was recovered by GPX1 overexpression, as observed in the results of CCK-8, EdU and colony formation experiments. (**E–G**) The enhanced GPX1 lessened the apoptotic ability of Molt-3 cells induced by VPS9D1-AS1 knockdown, as demonstrated by flow cytometry analysis, detection of caspase-3/9 activity and Western blotting. All assays were run in triplicate. *P<0.05 and **P<0.01.

To further validate whether GPX1 mediated the impact of VPS9D1-AS1 on ALL cellular function, we then performed rescue assays in VPS9D1-AS1-silenced Molt-3 cells after GPX1 expression was enlarged (Figure 4A and Supplementary Figure S3A). As a result, the restrained cell viability by silenced VPS9D1-AS1 was reversed under GPX1 overexpression (Figure 4B). The results of EdU experiment indicated that EdU positive cells were lowered with VPS9D1-AS1 down-regulation but recovered upon further up-regulation of GPX1 (Figure 4C). Likewise, the number of colonies was decreased by decline of VPS9D1-AS1, while such phenomenon was reversed under GPX1 enhancement (Figure 4D). Flow cytometry analysis showed that the stimulated apoptosis in VPS9D1-AS1-inhibited Molt-3 cells was rescued after overexpressing GPX1 (Figure 4E). Consistently, VPS9D1-AS1 silence-enhanced caspase-3/9 activities and cleaved caspase-3/9 levels were normalized in face of GPX1 overexpression (Figure 4F,G and Supplementary Figure S3B). In sum, VPS9D1-AS1 exerted a tumor-facilitating function in ALL via a GPX1-mediated way.

## Discussion

In TCGA website, the expression of GPX1 was assumed to be significantly high in LAML tissues. Previous researches have reported the oncogenic role of GPX1 in several carcinomas. For example, Liu et al. demonstrated that glutathione peroxidases have distinct prognostic values for non-small cell lung carcinoma [24]. Chen et al. indicated that GPX1 promotes NSCLC cisplatin-resistance via ROS-induced PI3K/AKT pathway [25]. Zhang et al. reported that overexpressed GPX1 indicates discontent survival in laryngeal squamous cell cancer [26]. In this study, GPX1 was observed to be dramatically up-regulated in ALL and loss-of-function assays revealed that inhibiting GPX1 restrained cell proliferation and promoted cell apoptosis in ALL.

Over the past years, miRs have been illustrated to interact with the 3′UTRs of mRNAs to silence these mRNAs [27,28]. Herein, miR-491-5p and miR-214-3p were recognized to target GPX1 in ALL. As past reports elucidated, miR-491-5p and miR-214-3p exerted tumor-inhibiting functions in diverse cancers [29,30]. FOXI1-mediated miR-491-5p serves as a tumor repressor in gastric cancer via targeting Wnt3a/β-catenin pathway [31]. MiR-491-5p induces cell apoptosis in ovarian cancer through direct inhibition of both BCL-XL and EGFR [32]. MiR-214-3p inhibits cell proliferation and cell cycle distribution via targeting MELK in hepatocellular carcinoma [33]. PVT1 knockdown hinders cell proliferation and invasion in colorectal cancer due to up-regulated miR-214-3p [12]. The interaction between miR-491-5p (or miR-214-3p) and GPX1 was first exposed by this work.

In last several decades, ceRNA hypothesis has been highly suggested in cancer [34]. LncRNAs are well-established molecules that are involved in a ceRNA network to affect miRs–mRNAs interaction to function in multiple cancers [35]. In current study, VPS9D1-AS1 was acknowledged as the most feasible lncRNA participating in miR-419-5p-miR-214-3p/GPX1 axis. The oncogenic role of VPS9D1-AS1 in malignancies has been unveiled by several papers. For examples, VPS9D1-AS1 (also called MYU) boosts prostate cancer cell proliferation by mediation of miR-184c-Myc axis [21]; increased expression of VPS9D1-AS1 predicts dismal prognosis in non-small cell lung cancer [22]. Consistently, our work also validated the contributing effect of VPS9D1-AS1 on the proliferation of ALL cells through an miR-491-5p-miR-214-3p/GPX1-mediated manner.

All in all, these above data elucidated that VPS9D1-AS1 elevated GPX1 in ALL by sequestering miR-491-5p and miR-214-3p, which may provide promising biomarkers for the therapy of ALL patients.

## Supplementary Material

Supplementary Figures S1-S3 and Supplementary File S1

## Abbreviations

Ago2: argonaute 2
ALL: acute lymphoblastic leukemia
BCL-XL: B-cell lymphoma-extra large
CCK-8: cell counting kit-8
ceRNA: competing endogenous RNA
DAPI: 4′6-diamidino-2-phenylindole
EDU: 5-Ethynyl-2′-deoxyuridine
EGFR: epidermal growth factor receptor
FISH: fluorescence in situ hybridization
FOXI1: forkhead box i1
GAPDH: glyceraldehyde-3-phosphate dehydrogenase
GPX1: glutathione peroxidase 1
IGF2BP1: insulin like growth factor 2 mRNA binding protein 1
LAML: acute myeloid leukemia
lncRNA: long noncoding RNA
MiR: microRNA
mRNA: messenger RNA
PBMC: peripheral blood mononuclear cell
PVT1: plasmacytoma variant translocation 1
qRT-PCR: quantitative real-time PCR
RIP: RNA immunoprecipitation
ROS: reactive oxygen species
TCGA: The Cancer Genome Atlas
VPS9D1-AS1: VPS9 domain-containing protein 1 antisense RNA 1
ZEB1: zinc finger E-box binding homeobox 1
