# Supplementary material for: LncRNA VPS9D1-AS1 promotes cell proliferation in acute lymphoblastic leukemia through modulating GPX1 expression by miR-491-5p and miR-214-3p evasion
Source: Biosci Rep. 2020 Oct 5;40(10):BSR20193461. doi: 10.1042/BSR20193461 (PMC7536331; doi:10.1042/BSR20193461)
Supplement: Supplementary Figures S1-S3 and Supplementary File S1 [file BSR-2019-3461_supp.pdf]

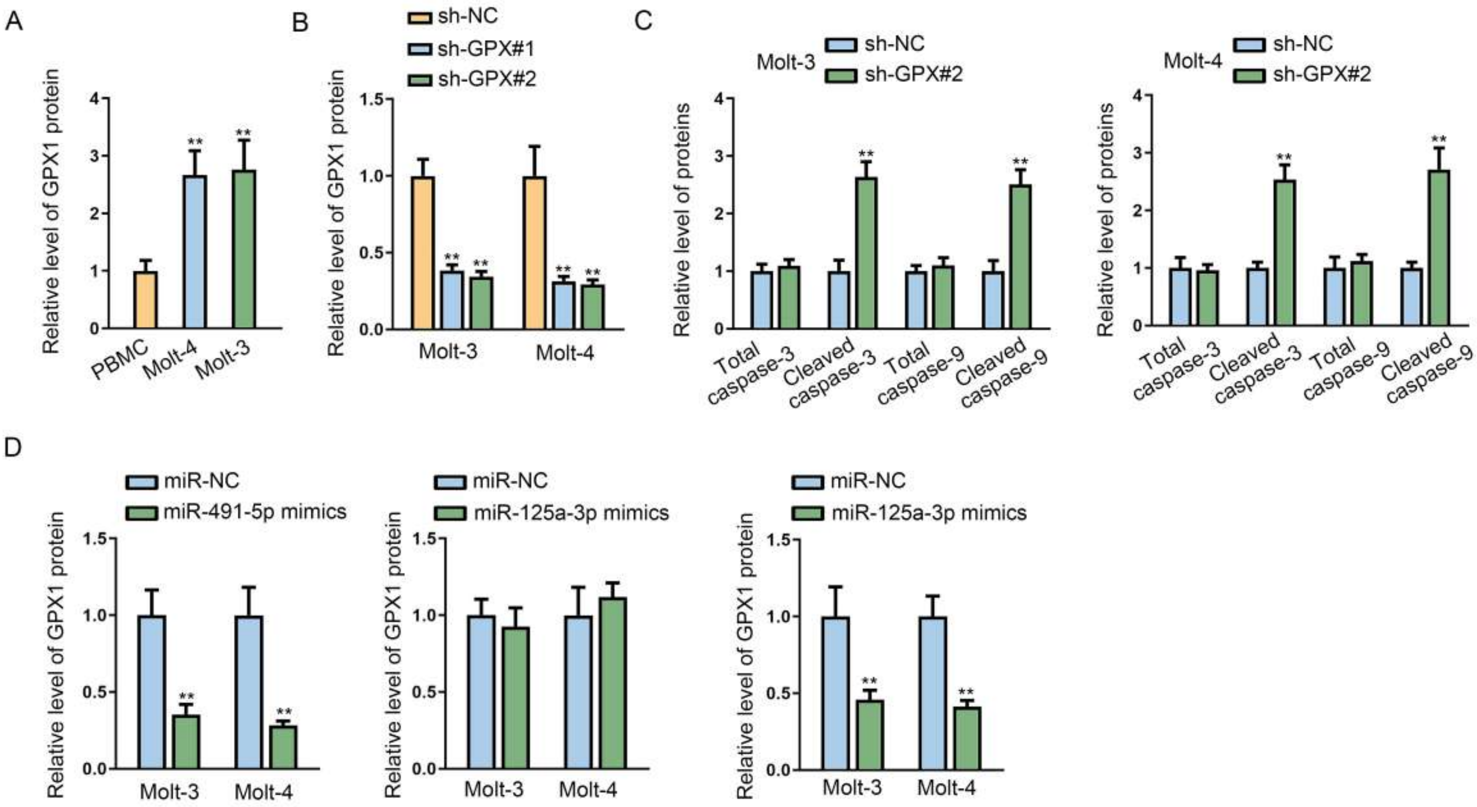

A
Relative level of GPX1 protein
PBMC
Molt-4
Molt-3
B
sh-NC
sh-GPX#1
sh-GPX#2
Relative level of GPX1 protein
Molt-3
Molt-4
C
Molt-3
sh-NC
sh-GPX#2
Relative level of proteins
Total caspase-3
Cleaved caspase-3
Total caspase-9
Cleaved caspase-9
Molt-4
sh-NC
sh-GPX#2
Relative level of proteins
Total caspase-3
Cleaved caspase-3
Total caspase-9
Cleaved caspase-9
D
miR-NC
miR-491-5p mimics
Relative level of GPX1 protein
Molt-3
Molt-4
miR-NC
miR-125a-3p mimics
Relative level of GPX1 protein
Molt-3
Molt-4
miR-NC
miR-125a-3p mimics
Relative level of GPX1 protein
Molt-3
Molt-4

Supplementary Figure 1. A-D. Quantitative histogram of western blots in Figures 1B (A), 1C (B), 1I (C) and 2E (D), respectively. $^{**}P < 0.01$.

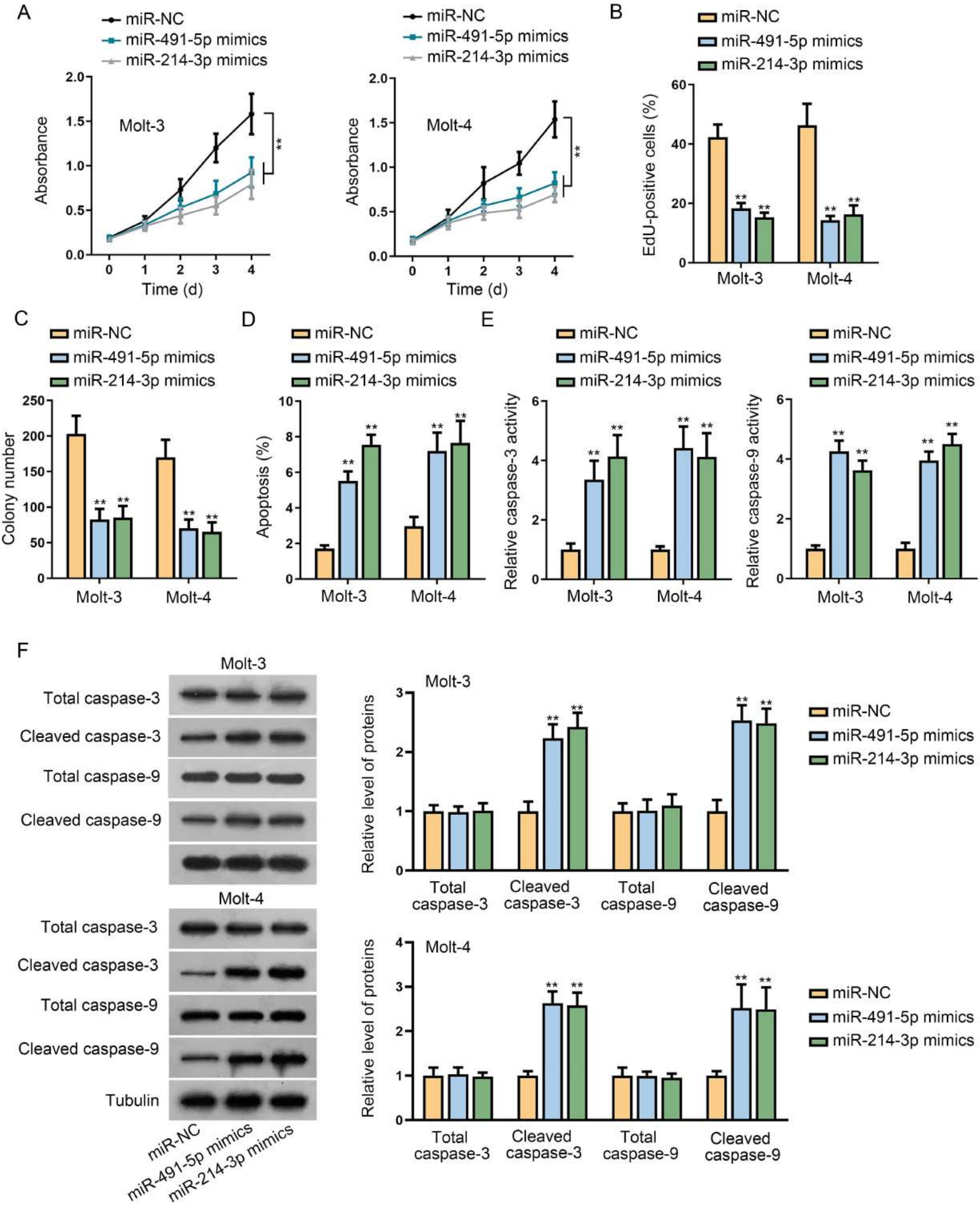

A
miR-NC
miR-491-5p mimics
miR-214-3p mimics
Absorbance
Molt-3
Molt-4
Time (d)
**
B
EdU-positive cells (%)
C
Colony number
D
Apoptosis (%)
E
Relative caspase-3 activity
Relative caspase-9 activity
F
Total caspase-3
Cleaved caspase-3
Total caspase-9
Cleaved caspase-9
Tubulin
Relative level of proteins

Supplementary Figure 2. A. The viability of ALL cells with overexpressed miR-491-5p or miR-214-3p was detected by CCK-8 assay. B-C. The impact of overexpressed miR-491-5p or miR-214-3p on the proliferation of ALL cells was determined via EdU and colony formation assays. D-E. Cell apoptosis under above conditions was assessed by flow cytometry analysis and caspase-3/9 activity detection. F. Western blot examined the level of several apoptosis-related proteins, with corresponding quantitative histograms beside. $^{**}P < 0.01$.

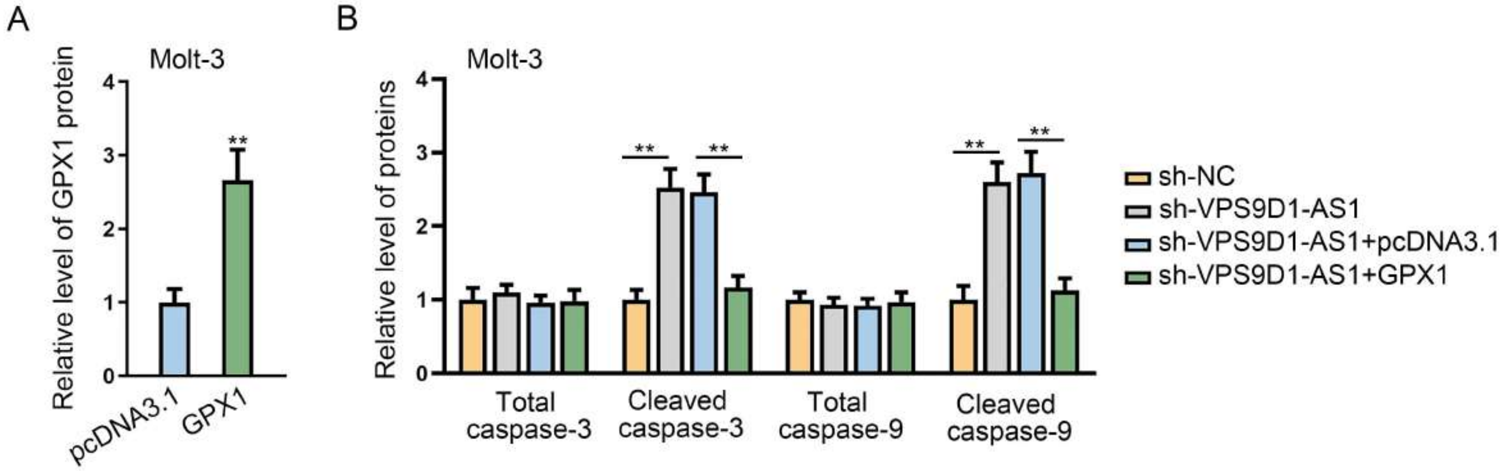
A
Molt-3
Relative level of GPX1 protein
4
3
2
1
0
**
pcDNA3.1
GPX1
B
Molt-3
Relative level of proteins
4
3
2
1
0
**
**
**
**
Total caspase-3
Cleaved caspase-3
Total caspase-9
Cleaved caspase-9
sh-NC
sh-VPS9D1-AS1
sh-VPS9D1-AS1+pcDNA3.1
sh-VPS9D1-AS1+GPX1

Supplementary Figure 3. A. The bar graph of quantified bands in Figure 4A. B. The bar graph of quantified bands in Figure 4G. $^{**}P < 0.01$.

A

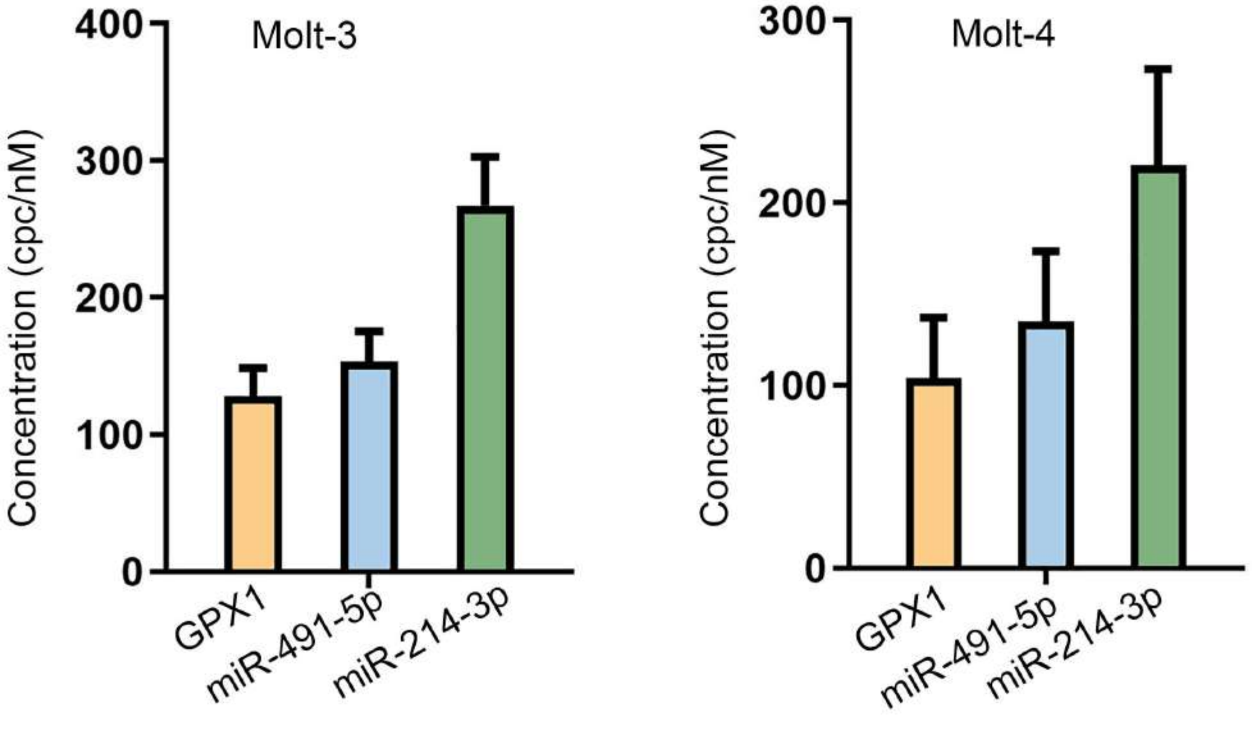

Molt-3
Concentration (cpc/nM)
400
300
200
100
0
GPX1
miR-491-5p
miR-214-3p
Molt-4
Concentration (cpc/nM)
300
200
100
0
GPX1
miR-491-5p
miR-214-3p

Supplementary file 1. A. The concentration of GPX1, miR-491-5p and miR-214-3p in Molt-3 and Molt-4 cells was determined using External RNA Controls Consortium RNA spike-in standards (Life Technologies).
